# Editorial: RNA Splicing and Backsplicing: Disease and Therapy

**DOI:** 10.3389/fgene.2020.626835

**Published:** 2020-12-08

**Authors:** Rosanna Asselta, Stefano Duga, Eladio Andrés Velasco, Emanuele Buratti

**Affiliations:** ^1^Department of Biomedical Sciences, Humanitas University, Milan, Italy; ^2^Humanitas Clinical and Research Center, Istituto di Ricovero e Cura a Carattere Scientifico, Milan, Italy; ^3^Splicing and Genetic Susceptibility to Cancer, Instituto de Biología y Genética Molecular, Valladolid, Spain; ^4^Consejo Superior de Investigaciones Científicas (CSIC-UVa), Valladolid, Spain; ^5^International Centre for Genetic Engineering and Biotechnology (ICGEB), Trieste, Italy

**Keywords:** splicing, backsplicing, RNA processing, circRNA, therapy

Splicing has been extensively studied in recent years both under physiological and pathological conditions. In particular, high-throughput RNA sequencing has allowed a much deeper knowledge on the breadth of alternative splicing in gene expression regulation. Besides the multiplicity of transcripts originating from “conventional” linear splicing, an additional layer of complexity is provided by backsplicing, mostly occurring at annotated exon boundaries, which produces circular RNAs (circRNAs). These are covalently closed RNA rings particularly stable compared to their linear counterparts because they are resistant to exonucleolytic decay; besides being potential therapeutic agents and targets, they also represent attractive biomarkers. In parallel, the increasing data from human genomes are providing, for the first time, population-wide landscapes of genetic variants potentially impacting on splicing, both in monogenic and complex diseases.

Splicing alterations have been particularly studied in autoimmune diseases, where they can be responsible for the generation of autoantigens, and in cancer, where the neo-antigens originating from splicing derangement can impact on tumor immunogenicity and have important consequences on the efficacy of immunotherapy. Moreover, the generation of alternatively spliced isoforms was shown to be implicated in drug resistance to chemotherapy. As a result, a number of approaches were developed to modulate splicing both to correct splicing mutations and to promote/silence specific splicing events as potential therapies, including antisense oligonucleotides, modified U1 snRNAs, small molecules acting on splicing, and trans-splicing. These post-transcriptional interventions have substantial advantages over traditional gene therapy, including no need to deliver large DNA constructs and no concerns regarding the tissue specificity and expression level of the transgene.

In view of these many implications in human diseases and the tremendous efforts aimed at translating the molecular understanding of splicing into the clinic, the purpose of this Research Topic was to provide an overview on new data on RNA splicing and backsplicing in disease and therapy. The articles in this topic range from understanding the mechanisms of splicing regulation, to evaluating the impact of splicing isoforms and circRNA detection on disease diagnosis and prognosis, up to possible therapeutic applications of splicing correction.

## Splicing Regulation and its Impact on Gene Expression

Despite decades of molecular studies, the splicing machinery is still only partially understood in its fine mechanistic details, and the work by Sebbag-Sznajder et al. points to the role of dynamic supraspliceosomes in the splicing regulation of all pre-mRNAs. These gigantic structures (21 MDa) package RNApol II transcribed pre-mRNAs into complexes composed of four native spliceosomes connected by the transcript. Supraspliceosomes also contain pre-mRNA processing factors (e.g., cap-binding proteins, 3′-end processing components, and the ADAR1 and ADAR2 editing enzymes). The supraspliceosome thus emerges as a principal regulator of multiple pre-mRNA processing steps (Sebbag-Sznajder et al.).

Concerning specifically the molecular mechanisms involved in splicing regulation, it has been recently demonstrated that intragenic elements within pre-mRNA can promote RNA structures that potently activate the RNA-dependent protein kinase PKR, which in turn induces nuclear eIF2α phosphorylation and thereby strongly enhances mRNA splicing efficiency. This mechanism has been extensively studied for the tumor necrosis factor-α gene and for fetal/adult globin genes. Knowledge of these regulatory mechanisms is crucial to interpret the functional consequences of genetic variations mapping in these intragenic regulatory elements. This may eventually help explaining the impact on splicing of numerous human β-thalassemia mutations (Kaempfer et al.).

Splicing regulation can also have dramatic effects on the downstream events, as exemplified by the alternative splicing event involving exon 2 of Bcl-x, which can result in the production of the anti-apoptotic Bcl-xL or the pro-apoptotic Bcl-xS isoforms. The control of the Bcl-xL/Bcl-xS splicing ratio, reviewed by Stevens and Oltean, is provided by serine/arginine-rich (SR) proteins, heterogeneous nuclear ribonucleoproteins (hnRNPs), transcription factors, and cytokines, and represents a clear example of the importance of splicing modulation (Stevens and Oltean).

## Annotation and Functional Interpretation of Variants Impacting on Splicing

A precise knowledge on the mechanism of splicing is also essential for the functional annotation of variants in order to correctly interpret genetic variation and its relationship with human diseases. Regarding this issue, two reviews in our Topic address the importance to incorporate an analysis on the consequences of genetic variations on RNA metabolism to improve clinical diagnosis (Marco-Puche et al.; Lye et al.). Indeed, it is estimated that RNA sequencing would increase the diagnostic rate by up to 10–35%, even though the dynamic nature of the transcriptome, which changes according to tissue type, cellular conditions, and environmental factors, makes this approach more complicated than classic DNA sequencing. Significant advances are being made in bioinformatics to define, homogenize, and monitor the transcriptomic information in order to endure reproducibility and repeatability, which are mandatory for clinical utility of these data. Lye et al. propose the introduction of RNAseq-based analysis for patients who have a clinical presentation of primary immunodeficiency, but despite having undergone whole-exome/whole-genome sequencing, remain undiagnosed (Lye et al.). Thompson et al. investigated the contribution of splicing-assay data to the classification of mismatch-repair (MMR) gene sequence variants (Thompson et al.).

An important aspect when considering the impact of genetic variation on RNA metabolism is how we validate and classify variants. In this specific area, three manuscripts of our Research Topic address this problem in relation to osteogenesis imperfecta and to the risk of breast cancer, providing useful information on the consequences of atypical splicing variants and improving the clinical interpretation of variants of *COL1A1/COL1A2* and *BRCA2* genes through systematic functional assays in splicing reporter minigenes (Li et al.; Fraile-Bethencourt et al.). Likewise, Walker et al. showed that the comprehensive assessment of alternative splicing events of the breast/ovarian cancer gene BARD1 is a valuable strategy for the classification of spliceogenic and truncating variants (Walker et al.).

Mucaki et al. evaluated the quality of *in-silico* predictions by information-theory-based variant analysis on the effect of nucleotide substitutions detected in different cancers on splicing. In this study, RT-PCR and RNAseq data confirmed in most cases predictions even though not all events were detected by both techniques stressing the importance of multiple approaches in functional validation.

## Circrnas as Biomarkers in Human Diseases

In recent years, there has been a growing interest in circRNAs as potential players in human diseases, especially cancer, and as useful clinical diagnostic markers. In this frame, a comprehensive circRNA profiling, described in the work of Kong et al., led to the discovery of cancer-specific circRNAs in gastric cancer.

Even though in most cases circRNAs are considered the product of backsplicing, at least a fraction of them were shown to originate from the transcription of circular DNAs of chromosomal origin, as discussed in the review by Iparraguirre et al.

A role for circRNAs in different types of treatment resistance has also emerged, and the mini-review from Jeyaraman et al. focuses on the possible role of circRNAs as regulators of treatment resistance in human cancers based on their regulatory role on specific cancer-related networks (Jeyaraman et al.).

Finally, an extensive dysregulation of circRNA biogenesis, leading a global increase in circRNA levels, was found in patients with myotonic dystrophy and circRNA levels were found to be associated with muscle weakness and alternative-splicing changes (Czubak et al.).

## Therapeutic Approaches Exploiting Splicing Correction

The precise knowledge on the mechanisms of splicing in physiology and disease has been instrumental to design novel therapeutic strategies for splicing-derived pathologies, which are nicely reviewed by Suñé-Pou et al. In their review, they focus on nanotechnology-based gene delivery strategies to overcome the challenges and barriers facing nucleic acid-based therapeutics, which still represent a major obstacle to the clinical translation of splicing-correction approaches (Suñé-Pou et al.).

Among the different strategies explored to correct splicing, the use of modified spliceosomal U1snRNAs has demonstrated to successfully correct splicing mutations in several cellular and mouse models of human disease. Concerning coagulation factor VIII, a number of splicing defects leading to hemophilia A were analyzed by a mini-gene expression approach to experimentally determine the best modified U1snRNA variant able to correct multiple hemophilia A-causing mutations by re-directing the use of the proper 5′ splice site (Balestra et al.).

Interfering with splicing for therapeutic purposes does not only involve restoring aberrant splicing but, in some cases, inducing aberrant splicing (exon skipping) can be exploited to exclude a mutated in-frame exon from the mature transcript. This is the case of the *DMD* gene that, when mutated, is responsible for Duchenne (DMD) and Becker (BMD) muscular dystrophies. The observation that most mutations producing internally truncated but partially functional protein are associated with the milder BMD phenotype suggested the idea to induce targeted exon skipping as a treatment strategy for severe DMD. This has been obtained using antisense oligonucleotides (AO) designed to anneal to splicing sites. Different strategies to improve AO potency have been explored, in order to improve cellular uptake or increase stability and specificity of AOs, such as the use of 2′-*O*-methyl (2′-OMe) or locked nucleic acid (LNA), which increase binding affinity and resistance against nuclease degradation. In their work, Zaw et al., by testing a set of AOs targeting exons 16, 23, and 51 of human *DMD*, showed that the incorporation of LNAs into 2′-OMe antisense sequences increases their potency as steric blockers of splicing. However, this increased potency came at the price of the activation of alternative cryptic splice sites, suggesting the need to carefully check for possible off-target effects when using this exon-skipping inducing molecules (Zaw et al.).

In summary, the articles presented in this Research Topic illuminate several aspects of RNA splicing and backspacing processes, ranging from their role in the regulation of gene expression, up to their involvement in diseases and related therapeutics/biomarkers. The next few years promise to shed further light on these aspects. In particular, we expect to have a comprehensive catalog of alternative-splicing/backsplicing genetic variants in different human populations, along with expression levels of the associated relevant genes (i.e., a complete catalog of splicing quantitative trait loci, sQTLs, as well as circQTLs in different tissues). This will be instrumental for improving both disease diagnosis and patient treatments, giving even more added value for advocating a precision medicine approach.

## Author Contributions

All authors listed have made a substantial, direct and intellectual contribution to the work, and approved it for publication.

## Conflict of Interest

The authors declare that the research was conducted in the absence of any commercial or financial relationships that could be construed as a potential conflict of interest.

